# *CcpA*-Knockout *Staphylococcus aureus* Induces Abnormal Metabolic Phenotype via the Activation of Hepatic STAT5/PDK4 Signaling in Diabetic Mice

**DOI:** 10.3390/pathogens12111300

**Published:** 2023-10-30

**Authors:** Yilang Li, Jiaxuan Cai, Yinan Liu, Conglin Li, Xiaoqing Chen, Wing-Leung Wong, Wenyue Jiang, Yuan Qin, Guiping Zhang, Ning Hou, Wenchang Yuan

**Affiliations:** 1 Guangzhou Key Laboratory for Clinical Rapid Diagnosis and Early Warning of Infectious Diseases, KingMed School of Laboratory Medicine, Guangzhou Medical University, Guangzhou 511436, China; yilangli@stu.gzhmu.edu.cn; 2 Guangzhou Municipal and Guangdong Provincial Key Laboratory of Molecular Target & Clinical Pharmacology, the NMPA and State Key Laboratory of Respiratory Disease, School of Pharmaceutical Sciences and the Fifth Affiliated Hospital, Guangzhou Medical University, Guangzhou 511436, China; 344034520@163.com (J.C.); 2020118118@stu.gzhmu.edu.cn (Y.L.); chenxiaoqing@stu.gzhmu.edu.cn (X.C.); guoqinyuan@126.com (Y.Q.); 1984990005@gzhmu.edu.cn (G.Z.); 3 The State Key Laboratory of Chemical Biology and Drug Discovery, Department of Applied Biology and Chemical Technology, The Hong Kong Polytechnic University, Hung Hom, Kowloon, Hong Kong 999077, China; wing.leung.wong@polyu.edu.hk; 4 The Sixth Affiliated Hospital of Guangzhou Medical University, Qingyuan People’s Hospital, Qingyuan 511518, China; jiangwy@gzhmu.edu.cn

**Keywords:** diabetes mellitus, catabolite control protein A, pyruvate dehydrogenase kinase 4, signal transducer and activator of transcription 5, metabolic phenotype

## Abstract

Catabolite control protein A (CcpA), an important global regulatory protein, is extensively found in *S. aureus*. Many studies have reported that CcpA plays a pivotal role in regulating the tricarboxylic acid cycle and pathogenicity. Moreover, the *CcpA*-knockout *Staphylococcus aureus* (*S. aureus*) in diabetic mice, compared with the wild-type, showed a reduced colonization rate in the tissues and organs and decreased inflammatory factor expression. However, the effect of *CcpA*-knockout *S. aureus* on the host’s energy metabolism in a high-glucose environment and its mechanism of action remain unclear. *S. aureus*, a common and major human pathogen, is increasingly found in patients with obesity and diabetes, as recent clinical data reveal. To address this issue, we generated *CcpA*-knockout *S. aureus* strains with different genetic backgrounds to conduct in-depth investigations. In vitro experiments with high-glucose-treated cells and an in vivo model study with type 1 diabetic mice were used to evaluate the unknown effect of *CcpA*-knockout strains on both the glucose and lipid metabolism phenotypes of the host. We found that the strains caused an abnormal metabolic phenotype in type 1 diabetic mice, particularly in reducing random and fasting blood glucose and increasing triglyceride and fatty acid contents in the serum. In a high-glucose environment, *CcpA*-knockout *S. aureus* may activate the hepatic STAT5/PDK4 pathway and affect pyruvate utilization. An abnormal metabolic phenotype was thus observed in diabetic mice. Our findings provide a better understanding of the molecular mechanism of glucose and lipid metabolism disorders in diabetic patients infected with *S. aureus*.

## 1. Introduction

Diabetes is a chronic disease generally caused by a dysfunctional pancreas that does not produce enough insulin or by the body’s ineffective use of the insulin produced. Both issues result in an increase in plasma glucose levels. Insufficient insulin secretion causes glucose metabolism disorders, such as reduced glucose entering the cells, reduced glycogen synthesis, weakened tricarboxylic acid (TCA) circulation, glucose accumulation in the blood, and reduced glucose utilization in the liver, muscle, fat, and other tissues. Along with these changes, the muscular and hepatic glycogen breakdown diminishes and gluconeogenesis increases, resulting in an increase in glucose production, leading to hyperglycemia. In addition, diabetes is often accompanied by lipid metabolic disorders, leading to multiple cardio-cerebrovascular diseases. Diabetes mellitus is often accompanied by dyslipidemia, which is a result of insulin resistance. Therefore, patients with diabetes who also have dyslipidemia need effective lipid-lowering treatment in addition to hypoglycemic therapy.

Depending on the different types of cells and oxygen supply, glucose catabolism mainly includes anaerobic oxidation, aerobic oxidation, and the pentose phosphate pathway. After high carbohydrate intake, blood glucose rises rapidly. It thus stimulates insulin secretion to promote cell uptake and the use of glucose. Moreover, insulin is the main hormone regulating fatty acid synthesis. By stimulating the activity of protein phosphatase, insulin dephosphorylates acetyl CoA carboxylase and promotes fatty acid synthesis. In addition, insulin promotes fatty acid synthesis and increases fat synthesis. In patients with diabetes, the ability of tissue cells to absorb glucose is decreased. The intracellular energy supply is thus insufficient. This may lead to increased hunger, increased eating, and an increased accumulation of glucose in the serum, consequently causing hyperglycemia [1]. Dyslipidemia caused by diabetes is closely related to insulin resistance, visceral obesity, and non-alcoholic fatty liver disease. In insulin-resistant individuals, excessive fatty acids flow into the liver, resulting in an excessive accumulation of very low-density lipoprotein [2]. Dyslipidemia caused by diabetes is typically a mixed hyperlipidemia, which is characterized moderately by increased low-density lipoprotein levels, increased total cholesterol levels, and decreased high-density lipoprotein levels [3].

Before taking insulin treatment into practice, bacterial infection was recognized as one of the most serious complications of diabetes mellitus, and it has been considered a critical cause of morbidity and mortality among diabetic patients. With the increased clinical use of insulin and antibiotics in treatment, the mortality rate of diabetes complicated with infection has reduced; nevertheless, the risk of diabetic patients dying from bacterial-related infections remains high. The reported standardized mortality rate is 1.83 [4]. *Staphylococcus aureus* (*S. aureus*) is an important human pathogen. Clinical infections related to *S. aureus* are a major global health problem and have been exacerbated by the emergence and spread of drug-resistant strains [5]. More importantly, recent evidence has revealed that the most predominant aerobic organism causing diabetic infections is *S. aureus*, accounting for 18.7% of cases [6]. In addition, *S. aureus* and MRSA colonization rates are approximately two times higher in diabetic patients than in patients without diabetes [7,8]. Therefore, *S. aureus* infections are most likely a crucial factor for the significant morbidity and mortality in patients with hyperglycemia [7]. This is probably attributed to the frequent complication of foot ulcers in diabetic patients [9]. It is thus critically important to obtain a deeper understanding of the molecular mechanism of *S. aureus* infection in patients with diabetes. Furthermore, some recent studies conducted in type 2 diabetic mice have shown that *S. aureus* infection induces insulin resistance, impairs glucose tolerance, and elevates blood glucose levels by secreting eLtaS protein, which binds to insulin [10]. Nonetheless, the exact effects of *S. aureus* infection on glucose and lipid metabolism and its molecular mechanism are not currently fully understood. A search for new molecular targets and innovative therapeutic strategies is thus urgently needed.

It is well-known that catabolite control protein A (CcpA) is a master regulator of carbon catabolite repression in *S. aureus* [11]. In the presence of glucose or other preferred carbon sources, CcpA forms a complex with S46-phosphorylated Hpr protein. The CcpA–Hpr-S46(P) complex binds with the catabolite-responsive element (*cre*) sequences of diverse genes to activate or repress their expression. CcpA represses the TCA cycle and is also instrumental in *S. aureus* pathogenesis [12,13,14]. In addition, the pyruvate dehydrogenase complex (PDC) is a multi-enzyme complex of the mitochondria and is implicated in glycolysis with the Krebs cycle. PDC is thus essential in producing acetyl CoA from glucose and regulates fuel consumption. The core PDC comprises three catalytic enzymes: pyruvate dehydrogenase (PDH), dihydrolipoamide acetyltransferase (DLAT), and dihydrolipoamide dehydrogenase (DLD). [15] The activity of PDC is regulated by pyruvate dehydrogenase kinases (PDKs). The main PDK subtypes expressed in the liver are PDK1, PDK2, and PDK4. PDK4 is a key regulator of PDC activity as well as pyruvate oxidation and glucose balance in vivo. It also inhibits PDC activity by phosphorylating PDH [16,17].

Considering the background information discussed above, we hypothesized that when *S. aureus* invades the body, CcpA protein may profoundly affect the glucose and lipid metabolism of the host in a high-glucose environment. In the present study, type 1 diabetic mice induced by streptozotocin injection and WRL68 human hepatocytes and subjected to high-glucose treatment were used as a model to investigate the potential effects of CcpA on abnormal metabolic phenotypes of diabetes. In addition, we studied the underlying mechanism for the first time to understand whether the phenotypes are associated with the activation of the liver’s signal transducer and activator of transcription 5 (STAT5)/PDK4 pathway. The study may provide significant insights into the development of antimicrobial agents targeting CcpA and their potential use in patients with diabetes.

## 2. Methods

### 2.1. Strains and Plasmids

The bacterial strains and plasmids used in this study are listed in Table 1. The *S. aureus* strain XN108 was isolated from a burn patient on 4 March 2004. RN4220 and Mu50 were kindly provided by Dr. Xiancai Rao (Army Medical University, China). The *S. aureus* Δ*CcpA* strain was constructed using an allelic replacement strategy as follows. Briefly, the *CcpA* gene was amplified by PCR from the genomic DNA of XN108 using the primer pairs pBT2–*CcpA*–5′ and pBT2–*CcpA*–3′. Then, it was cloned into the temperature-sensitive shuttle vector pBT2, which is widely used for markerless double homologous recombination. The pBT2–Δ*CcpA* plasmid was transformed into the *S. aureus* strain RN4220, extracted, purified, and then electro-transformed into XN108 to generate XN108 ΔCcpA after recombination. The Δ*CcpA* mutation after allelic exchange was confirmed by Sanger sequencing. The mutant Mu50 Δ*CcpA* was constructed using similar allelic replacement techniques.

### 2.2. Animals and the Establishment of Streptozotocin-Induced Diabetic Mice

The ethics committee of Guangzhou Medical University (Guangzhou, China) approved the protocols for the use and care of animals (approval number: GY2017–040). The handling and treatment of mice were conducted in strict accordance with Animal Research: Reporting of In Vivo Experiments (ARRIVE) guidelines for reporting experiments involving animals.

Adult male C57BL/6 mice (7–8 weeks, 20.0 g ± 2.0 g) were purchased from the Guangdong Medical Laboratory Animal Center (Guangzhou, China). Mice were housed in separate cages in a controlled environment (12-h/12-h day/night cycle, 50–70% humidity, and 26 °C temperature) and had ad libitum access to food and water. One-week acclimatization was performed to minimize stress.

Type 1 diabetes mellitus was induced by intraperitoneal injection of streptozotocin (Sigma-Aldrich, St. Louis, MO, USA) at a dose of 45 mg/kg/day for 5 days [18]. Age-matched male C57 mice were used as controls and given the same volume of citrate buffer. The level of plasma glucose was determined using a glucometer (OneTouch^TM^ Ultra Mini^@^ Blood Glucose Monitoring System, LLC, USA) three days after the final injection. Mice with a plasma glucose level of >16.7 mmol/L were defined as having DM.

### 2.3. Experimental Conditions for the Mice with Diabetes

Four weeks after DM induction, the mice were randomly divided into three groups: (1) the diabetic group (diabetic + vehicle, n = 8), which received intraperitoneal injections of PBS; (2) a diabetic group wherein the jugular vein was injected with 1 × 10^6^ CFU *S. aureus* XN108 Δ*CcpA* (diabetic + XN108 Δ*CcpA*, n = 9); and (3) a diabetic group wherein the jugular vein was injected with 1 × 10^6^ CFU wild-type *S. aureus* XN108 (diabetic + WT, n = 9). The control mice were randomly divided into three groups similar to those of the diabetic mice (non-diabetic + vehicle, non-diabetic + Δ*CcpA*, and non-diabetic + WT; n = 9 for each group). All mice were fed a normal diet and had ad libitum access to drinking water during the experiment.

### 2.4. Histological Measurements

Murine livers were fixed with 4% paraformaldehyde and immersed in paraffin. For hematoxylin and eosin and immunohistochemical staining [19], paraffin-embedded hepatic tissues from the mice in each group were cut into 4 μm sections using a microtome. After three cycles of deparaffinization in xylene and tissue rehydration, antigen retrieval was performed in Tris buffer (pH 9.0; Dako, Santa Clara, CA, USA) by heating to 99 °C for 20 min. The endogenous peroxidase activity was quenched with 3% H_2_O_2_, and non-specific binding was blocked with 10% non-immune goat serum (Life Technologies, Waltham, MA, USA); this was followed by the incubation of samples with primary antibodies at 4 °C overnight. TNF-α antibody and IL-6 antibody (Abcam, Cambridge, UK) were used at a 1:200 dilution. The signal was amplified using the Histostain SP Kit (Life Technologies, Waltham, MA, USA) and detected with diaminobenzidine (DAB) substrate (Dako, Santa Clara, CA, USA). The color development of DAB for diseased and control livers was microscopically monitored in parallel with the same reaction time. Hematoxylin was used as a counterstain. The intensity of TNF-α and IL-6 was quantified with the 3 sets of IHC straining images by Image J software (ImageJ bundled with 64-bit Java version 1.8.0_172). Negative controls were treated similarly, except for the omission of primary antibodies.

### 2.5. Determination of the Level of Random Plasma Glucose, Fasting Plasma Glucose, and Glucose Tolerance

The random plasma glucose level and fasting plasma glucose level were measured using a glucose analyzer (OneTouch Ultra Mini Blood Glucose Monitoring System, Johnson Co., New Brunswick, NJ, USA). Glucose tolerance was determined using intraperitoneal glucose tolerance tests (IPGTTs) in overnight-fasted mice [20]. After the intraperitoneal injection of glucose (2 g/kg), the plasma glucose level in blood samples obtained from the caudal vein was measured at 0, 30, 60, 90, and 120 min using the glucometer. The area under the blood glucose curve (AUC) was measured using the trapezoidal rule [21].

### 2.6. Transcriptome Sequencing

Liver tissues from twelve male C57BL/6 mice per group were dissected and immediately frozen in liquid nitrogen. Total RNA was extracted with TRIzol (Life Technologies, Waltham, MA, USA) reagent. RNA integrity (RNA integrity ≥ 7.9) and quantity were determined with an Agilent 2100 Bioanalyzer. Equal RNA amounts from individual mice were combined at 3 samples per pool, and 3 pools per group were analyzed in the Illumina HiSeqTM2000 at BGI Shenzhen (Beijing Genome Institute, Shenzhen 518083, China). All reads that passed quality metrics were mapped on the mouse transcriptome and genome using HISAT40/Bowtie241 tools. Then, normalization was performed with the mapped data, and the FPKM (fragments per kilobase per million mapped reads) was calculated using RESM software. An FDR (false discovery rate) < 0.01 and an absolute value of log2 Ratio ≥ 2 were used to identify differentially expressed genes (DEGs) in the diabetic + vehicle versus diabetic + XN108 ΔCcpA groups and the diabetic + vehicle versus diabetic + WT groups [22].

### 2.7. Cell Culture

WRL-68 cells were purchased from Guang Zhou Jennio Biotech Co., Ltd., Guangzhou, China. The cells were cultured in Dulbecco’s modified Eagle medium (DMEM) with L-glutamine (Gibco, Gland island, NE, USA) supplemented with 10% *v*/*v* fetal bovine serum (FBS; Gibco, Waltham, MA, USA) and the bacterial strain (1.5 × 10^7^ CFU/mL) at 37 °C and 5% CO_2_. The control group samples were cultured in DMEM with 10% *v*/*v* FBS.

### 2.8. Western Blotting

Hepatic protein samples and WRL68 cellular protein samples for Western blotting were extracted using 10× RIPA buffer (Cell Signaling, Danvers, MA, USA) supplemented with protease inhibitors. The following antibodies were purchased: rabbit anti-PDK1 (BS1291, Bioworld Technology, St Louis Park, MN, USA); rabbit anti-PDK2 (ab68164, Abcam, Cambridge, UK); rabbit anti-PDK4 (ab214938, Abcam, Cambridge, UK); rabbit anti-PDH (ab168379, Abcam, Cambridge, UK); rabbit anti-p-PDH (NB110-93479SS, NOVUS, Minneapolis, MN, USA); rabbit anti-PPARα (ab24509, Abcam, Cambridge, UK); rabbit anti-PGC-1α (ab191838, Abcam, Cambridge, UK); rabbit anti-FOXO1 (ab52857, Abcam, Cambridge, UK); rabbit anti-p-FOXO1 (9461, Cell Signaling, Danvers, MA, USA); rabbit anti-STAT5 (94205, Cell Signaling, Danvers, MA, USA); rabbit anti-p-STAT5 (ab32364, Abcam, Cambridge, UK); rabbit anti-β-actin (AP0060, Bioworld Technology, Minnesota, USA); and anti-rabbit IgG HRP-linked antibody (7074P2, Cell Signaling, Danvers, MA, USA). Herein, 1 μg/mL primary antibodies and 50 ng/mL secondary antibodies were used for blotting.

For Western blotting, the membrane was blocked with 5% skim milk in PBST for 1 h at 25 °C with gentle shaking. Next, the membrane was incubated with primary antibodies diluted in a solution containing 3% BSA in PBST overnight at 4°C with gentle shaking. Furthermore, the membrane was washed three times for 10 min in PBST with vigorous shaking. Then, at 25 °C, the membrane was incubated with secondary antibodies diluted in 5% skim milk in PBST for 1 h with gentle shaking. The membrane was again washed three times for 10 min in PBST with vigorous shaking. Detection was performed using SuperSignal^TM^ West Pico PLUS (Thermo, Waltham, MA, USA) [23].

### 2.9. RNA Isolation and qRT-PCR

Total RNA from liver tissues was extracted using TRIzol (Life Technologies, USA). Complementary DNA (cDNA) was synthesized using the First Strand cDNA Synthesis Kit (Takara, Japan) according to the manufacturer’s instructions. RT-qPCR was performed in a 20 μL system using the QuantiTect SYBR Green PCR Kit (Takara, Osaka, Japan) on an ABI StepOne^TM^ Real-Time PCR System (Thermo Fisher Scientific, Waltham, MA, USA). The primer sequences used were as follows: *PDK1*, 5′-TTCCTGGACTTCGGGTCAGT-3′ (forward) and 5′-GTACGGATGGGGTCCTGAGA-3′ (reverse); *PDK2*, 5′-TTCAGCAAGTTCTCCCCGTC-3′ (forward) and 5′-GACATACCAGCTCTGCACCA-3′ (reverse); *PDK4*, 5′-AGCAGTAGTCGAAGATGCCTT-3′ (forward) and 5′-CACGATGTGGATTGGTTGGC-3′ (reverse); *Pdhα1*, 5′-GGGACGTCTGTTGAGAGAGC-3′ (forward) and 5′-TGTGTCCATGGTAGCGGTAA-3′ (reverse); and *Glc6p*, 5′-CTCTGGGTGGCAGTGGTCGG-3′ (forward) and 5′-AGGACCCACCAATACGGGCGT-3′ (reverse).

### 2.10. Measurement of Biochemical Indicators

Biochemical indicators—the levels of total cholesterol, triglycerides, and glycated serum protein—were determined using diagnostic kits (Dade Behring Holdings, Shanghai, China) with an auto-analyzer (Siemens Healthcare Diagnostics, Nuremberg, Germany).

### 2.11. Measurement of the Content of Pyruvic Acid

Pyruvic acid content was determined using a commercial quantitative kit (Sigma-Aldrich, USA) according to the manufacturer’s instructions.

### 2.12. Statistical Analyses

Data are represented as the mean ± standard error of the mean. Differences between groups were analyzed using SPSS v18.0 (IBM, New York, NY, USA). One-way analysis of variance with Bonferroni post hoc testing was used for comparing multiple groups. A *p*-value of <0.05 was considered significant.

## 3. Results

### 3.1. The S. aureus XN108 ΔCcpA Strain Reduced Inflammatory Liver Injury in Diabetic Mice

**Figure 1:**
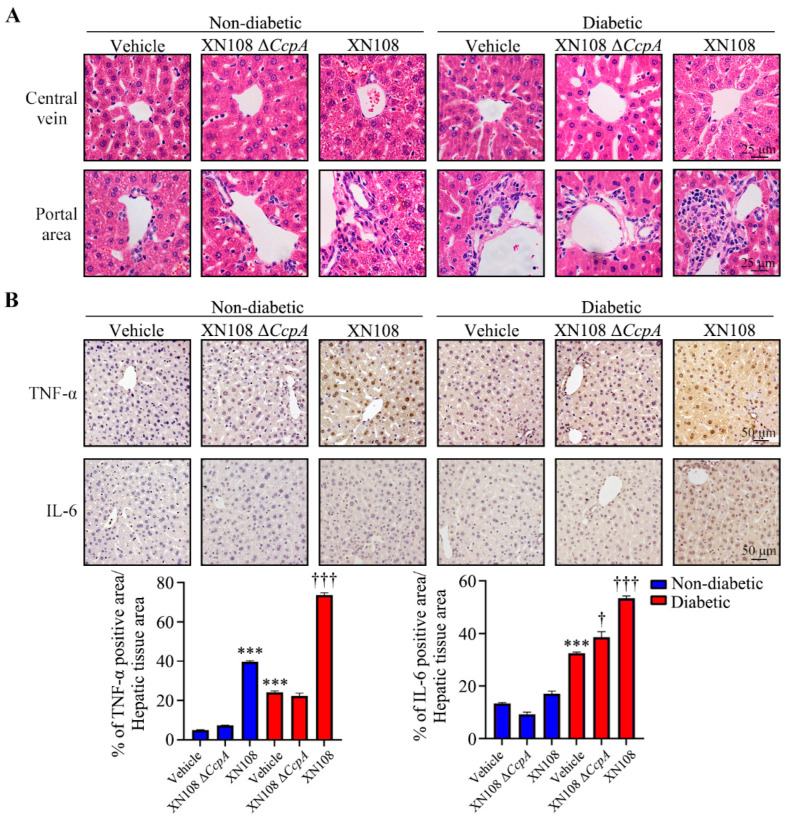
*S. aureus* XN108 Δ*CcpA* strain can reduce inflammatory liver injury. (**A**): Representative images of hematoxylin–eosin staining for liver cells around the central vein (**upper**) and portal area (**bottom**) of control and diabetic mouse livers infected with XN108 Δ*CcpA* or XN108 strain for 2 weeks; scale bar, 25 μm. (**B**): Representative images from three sets of immunohistochemical staining revealed expression level of TNF-α (**upper**) and IL-6 (**bottom**) and quantitative results of the positive area/hepatic tissue area ratio in the indicated groups; scale bar, 50 μm. *** *p* < 0.001 vs. non-diabetic + vehicle, † *p* < 0.05 vs. diabetic + vehicle, ††† *p* < 0.001 vs. diabetic + vehicle.

The result of liver tissue HE staining revealed that the liver tissue cells of the non-diabetic group had an ordered arrangement with uniform cell size and intercellular space. On the contrary, the arrangement of liver tissue cells in the diabetic group was relatively unstructured, with non-uniform cell size and morphology. In non-diabetic mice, the interstitial arrangement of liver tissues was more disorderly in bacteria-infected mice than in the uninfected mice; conversely, the liver cells were blurrier and more disorderly in the non-diabetic + XN108 group than in the non-diabetic + XN108 Δ*CcpA* group. In diabetic mice, the morphology and arrangement of the liver cells in the diabetic + XN108 Δ*CcpA* group were better than those in the diabetic group; however, hepatocytes in the diabetic + XN108 mice showed injury and edema, and numerous inflammatory cells were evident in the portal area (Figure 1A).

We also used immunohistochemistry to detect the expression of inflammatory factors in the liver tissues and found that the expression of TNF-α and IL-6 in hepatocytes was significantly increased in the non-diabetic + XN108 group and diabetic + XN108 group. However, the expression of inflammatory factors in the livers of normal and diabetic mice infected with the XN108 Δ*CcpA* strain and that of the control mice were comparable (Figure 1B).

### 3.2. Metabolic Phenotype in Type 1 Diabetic Mice Infected with XN108 ΔCcpA Strain Was Changed

**Figure 2:**
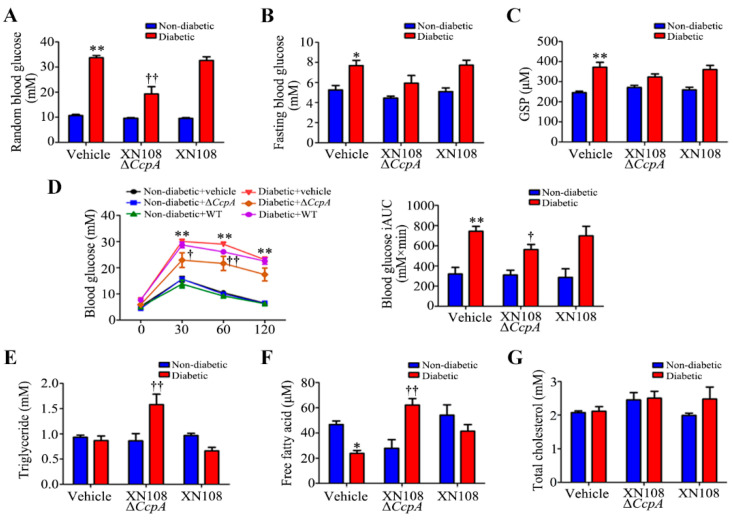
Changes in metabolic phenotype in type 1 diabetic mice after infection with the XN108 ΔccpA strain. (**A**): Quantitative results of random blood glucose level in control and T1DM mice infected with XN108 Δ*CcpA* or XN108 strain for 2 weeks. ** *p* < 0.01 vs. non-diabetic + vehicle; †† *p* < 0.01 vs. diabetic + vehicle; n = 5–6 mice per group. (**B**): Quantitative results of fasting blood glucose levels in the indicated groups. * *p* < 0.05 vs. non-diabetic + vehicle; n = 5–6 mice per group. (**C**): Quantitative results of the serum level of Glycated serum protein(GSP) in the indicated groups. ** *p* < 0.01 vs. non-diabetic+vehicle; n = 5–6 mice per group. (**D**): Intraperitoneal glucose tolerance test was performed in the indicated groups. The glucose tolerance curve and the mean incremental area under the glucose tolerance curve are shown. ** *p* < 0.01 vs.non-diabetic+vehicle; † *p* < 0.05 or †† *p* < 0.01 vs. diabetic+vehicle; n = 5–6 mice per group. (**E**): Quantitative results of the serum level of Triglyceride in the indicated groups. †† *p* < 0.01 vs. diabetic+vehicle; n = 5–6 mice per group. (**F**): Quantitative results of the serum level of Free fatty acid in the indicated groups. * *p* < 0.05 vs. non-diabetic+vehicle; †† *p* < 0.01 vs.diabetic+vehicle; n = 5–6 mice per group. (**G**): Quantitative results of the serum level of Total cholesterol in the indicated groups; n = 5–6 mice per group.

In the mice infected with *S. aureus* for two weeks, there was no significant change in the random blood glucose, fasting blood glucose, glycosylated serum protein levels, and glucose tolerance in the non-diabetic + XN108 Δ*CcpA* group or the non-diabetic + XN108 group, whereas the random blood glucose, fasting blood glucose, and glycosylated serum protein levels were significantly increased in the diabetic group. The AUC of the IPGTT glucose tolerance was measured for statistics; the AUC of the IPGTT was significantly increased in the diabetic group. Compared with the diabetic group, the random blood glucose and the AUC of the IPGTT were significantly decreased in the diabetic + XN108 Δ*CcpA* group (*p* < 0.05). Although the fasting blood glucose and glycosylated serum protein levels were decreased, the difference was not statistically significant (Figure 2A–D). In addition, the levels of triglycerides and free fatty acids in the diabetic + XN108 Δ*CcpA* group were significantly higher than those in the diabetic group (Figure 2E–G); however, there were no significant changes in the diabetic + XN108 group and the non-diabetic group in this regard.

### 3.3. XN108 ΔCcpA Strain Upregulated the PDK4 Expression in the Liver of Diabetic Mice

**Figure 3:**
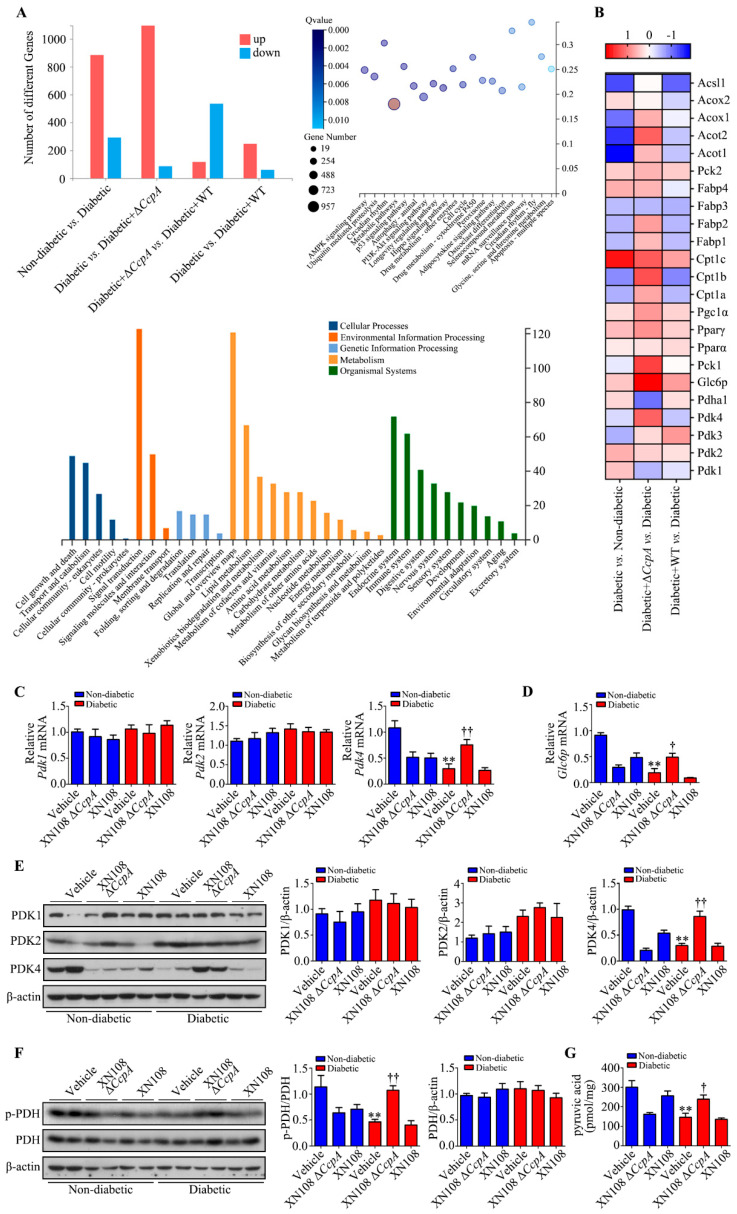
Effect of infection with the XN108 ΔCcpA strain on liver PDKs in diabetic mice. (**A**): Upregulated genes and downregulated genes are depicted in red and blue, respectively (n = 3/group). KEGG disease pathways and GO analysis of changed genes (*p* < 0.05). (**B**): Heatmaps depicting row Z-scores of FPKMs of upregulated and downregulated genes involved in lipid metabolism in the liver in the indicated groups. (**C**): Quantitative results of the relative mRNA levels of *PDK1, PDK2*, and *PDK4* in livers obtained from the indicated groups. ** *p* < 0.01 vs. non-diabetic + vehicle; †† *p* < 0.01 vs. diabetic + vehicle; n = 5–6 mice per group. (**D**): Quantitative results of the relative mRNA level of *Glc6p* in livers obtained from the indicated groups. ** *p* < 0.01 vs. non-diabetic + vehicle; † *p* < 0.05 vs. diabetic + vehicle; n = 5–6 mice per group. (**E**): Representative Western blots and quantitative results of the relative protein levels of PDK1, PDK2, and PDK4 in livers obtained from the indicated groups. ** *p* < 0.01 vs. non-diabetic + vehicle; †† *p* < 0.01 vs. diabetic + vehicle; n = 5–6 mice per group. (**F**): Representative Western blots and quantitative results of the relative protein levels of p-PDH and PDH in livers obtained from the indicated groups. ** *p* < 0.01 vs. non-diabetic + vehicle; †† *p* < 0.01 vs. diabetic + vehicle; n = 5–6 mice per group. (**G**): Quantitative results of the level of pyruvic acid in livers obtained from the indicated groups. ** *p* < 0.01 vs. non-diabetic + vehicle; † *p* < 0.05 vs. diabetic + vehicle; n = 5–6 mice per group.

The RNA-seq results showed that there were 1177 differentially expressed genes between the diabetic group and the non-diabetic group. In addition, there were 1182 differentially expressed genes between the diabetic + XN108 Δ*CcpA* group and the diabetic group; among these genes, 1076 genes were upregulated, and 86 genes were downregulated (Figure 3A). The gene expression signature of diabetic + XN108 ΔCcpA mice revealed differences in molecular functions related to ‘lipid and carbohydrate metabolism’ in the liver. Through Kyoto Encyclopedia of Genes and Genomes (KEGG) pathway enrichment analysis, we found that there were 957 metabolism-related genes (Figure 3A). After screening, PDK family genes were found to be the key regulatory genes of the metabolic phenotypic changes (Figure 3B).

The RNA-seq results verified using RT-PCR revealed that the gene expression of *PDK4* in the livers of the diabetic group was lower than that in the livers of the non-diabetic group, whereas the expression of *PDK4* in the livers of the diabetic + XN108 Δ*CcpA* group was significantly higher than that in the livers of the diabetic group. There was no significant difference in liver *PDK4* expression between the diabetic + XN108 group and the diabetic group. The expression of *PDK1* and *PDK2* did not significantly differ between groups (Figure 3C). *Glc6p* is a downstream gene regulated by PDK4. The expression of *Glc6p* in the livers of the diabetic + XN108 Δ*CcpA* group was significantly higher than that in the livers of the diabetic group (Figure 3D, *p* < 0.05), which was consistent with the differential PDK4 expression between groups (Figure 3C).

The Western blotting results showed that the expression of PDK4 protein in the livers of the diabetic + XN108 Δ*CcpA* group was significantly higher than that in the livers of the diabetic group; however, the expression of PDK1 and PDK2 showed no notable changes (Figure 3E). The expression of PDK4 at both the gene and protein levels was significantly increased in the livers of the diabetic + XN108 Δ*CcpA* group. We thus speculated that this could further affect the expression of downstream PDH. Examining the level of PDH phosphorylation in the diabetic + XN108 Δ*CcpA* group revealed that it was significantly higher than that in the control group (*p* < 0.01; Figure 3F). PDC catalyzes the irreversible oxidative decarboxylation of pyruvate to acetyl coenzyme A, which connects the aerobic oxidation of sugar with the TCA cycle. At the same time, PDH also participates in the first step of the catalytic reaction of PDC, in which pyruvate is decarboxylated to hydroxyethyl-TPP. As a result, the activity of the PDH catalytic reaction is directly correlated with the pyruvate content. Analyzing the pyruvate content in the livers of the mice revealed that the content in the diabetic group was significantly lower than that in the non-diabetic group. In addition, the pyruvate content in the diabetic + XN108 Δ*CcpA* group was significantly higher than in the diabetic + vehicle group (*p* < 0.05; Figure 3G). Taken together, the results obtained clearly showed that PDK4 expression was increased in the livers of mice in the diabetic + XN108 Δ*CcpA* group and that PDK4 upregulated the level of PDH phosphorylation, inhibited the activity of the PDC reaction, and enhanced pyruvate accumulation in the livers of the mice.

### 3.4. p-STAT5 was Involved in the Upregulation of PDK4 Expression in the Diabetic Mice Infected with the XN108 ΔCcpA Strain

**Figure 4:**
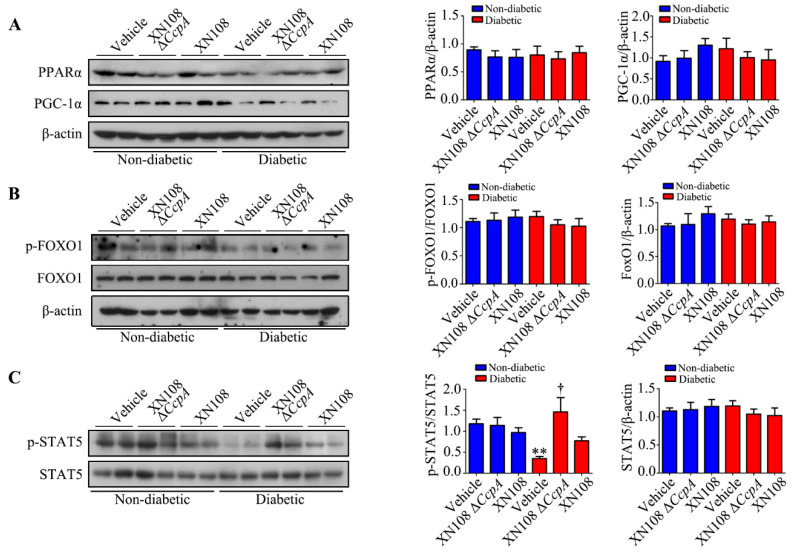
Upstream regulation mechanism of PDK4 in the livers of diabetic mice infected with the XN108 ΔccpA strain. (**A**): Representative Western blots and quantitative results of the relative protein levels of PPARα and PGC-1α in livers obtained from the indicated groups. (**B**): Representative Western blots and quantitative results of the relative protein levels of p-FOXO1 and FOXO1 in livers obtained from the indicated groups. (**C**): Representative Western blots and quantitative results of the relative protein levels of p-STAT5 and STAT5 in livers obtained from the indicated groups. ** *p* < 0.01 vs. non-diabetic + vehicle; † *p* < 0.05 vs. diabetic + vehicle; n = 5–6 mice per group.

The experimental results suggest that the XN108 Δ*CcpA* strain significantly upregulates the expression of PDK4 in the livers of diabetic mice. We thus further investigated the mechanism of PDK4 activation in the present study. Recently, various nuclear receptors and transcription factors involved in the regulation of PDK4 expression have been reported. Among them, PGC-1α, FOXO1, and ERRα are considered to be relevant to metabolism [17,24,25,26]. We thus investigated the key proteins of the above pathways with Western blotting. However, the results showed that there were no significant changes in the expression of PPARα, PGC-1α, or FOXO1 proteins (Figure 4A,B).

It was found that endogenous STAT5, after phosphorylation, is able to enter the nucleus and bind directly to the promoter of the PDK4 gene, subsequently promoting the expression of PDK4 [27]. Our results showed that the p-STAT5 in the diabetic group was significantly lower than that in the non-diabetic group. In addition, the p-STAT5 in the livers of the diabetic + XN108 Δ*CcpA* group was significantly higher than that in the livers of the diabetic group. More importantly, the trend of the changes was consistent with that of PDK4 (Figure 4C). The results indicate that p-STAT5 was most likely involved in the upregulation of PDK4 expression in the diabetic mice infected with the XN108 ΔCcpA strains.

### 3.5. Infection with S. aureus with Different Genetic Backgrounds Showed Different Effects on WRL68 Hepatocytes Induced by High Glucose

**Figure 5:**
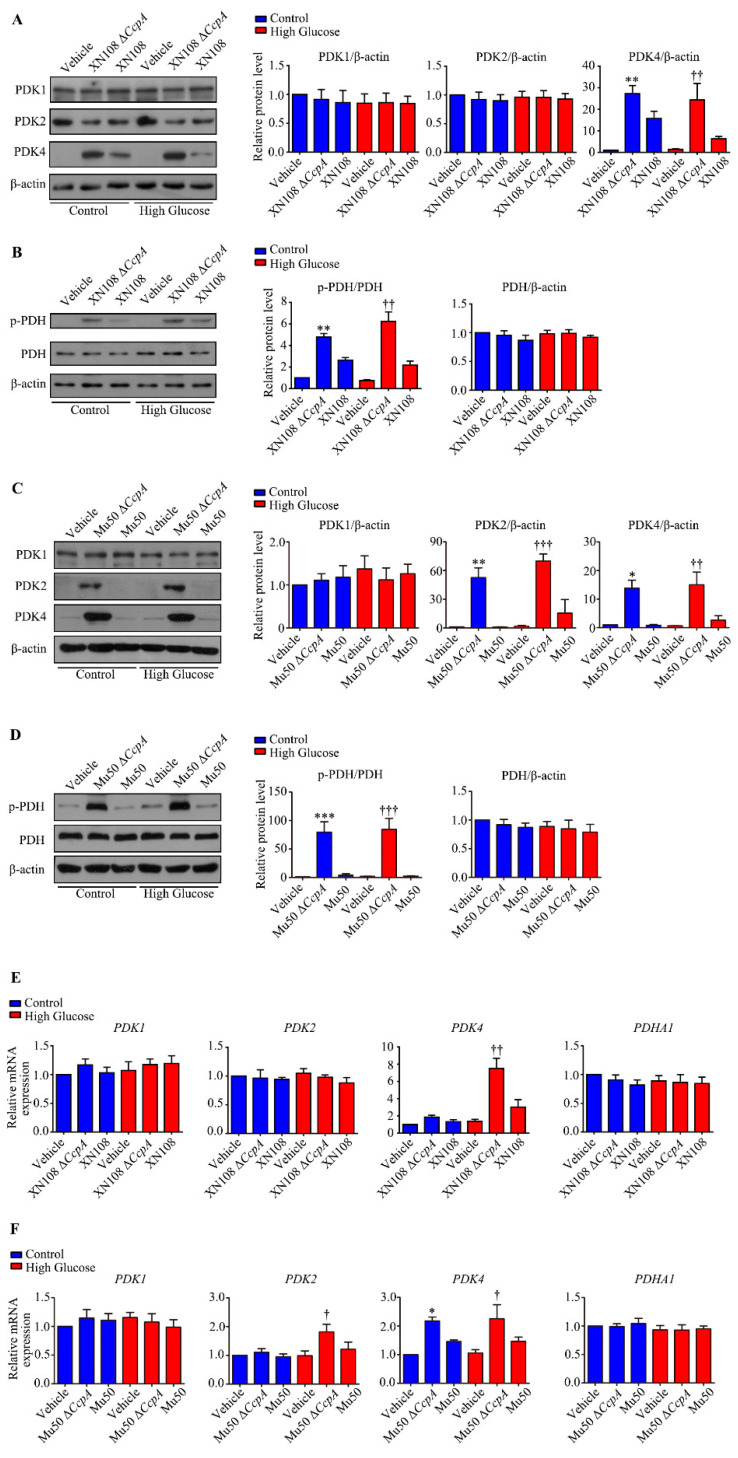
Effects of infection with *S. aureus* with different genetic backgrounds on WRL68 hepatocytes induced by high glucose. (**A**): Representative Western blots and quantitative results of the relative protein levels of PDK1, PDK2, and PDK4 in WRL68 cells subjected to high glucose (HG) with the XN108 Δ*CcpA* or XN108 strain for 4 h. ** *p* < 0.01 vs. control + vehicle; †† *p* < 0.01 vs. HG + vehicle. The above results are from five independent experiments. (**B**): Representative Western blots and quantitative results of the relative protein levels of p-PDH and PDH in WRL68 cells subjected to HG with the XN108 Δ*CcpA* or XN108 strain for 4 h. ** *p* < 0.01 vs. control + vehicle; †† *p* < 0.01 vs. HG + vehicle. The above results are from five independent experiments. (**C**): Representative Western blots and quantitative results of the relative protein levels of PDK1, PDK2, and PDK4 in WRL68 cells subjected to high glucose (HG) with the Mu50 Δ*CcpA* or Mu50 strain for 4 h. * *p* < 0.05 vs. control + vehicle; ** *p* < 0.01 vs. control + vehicle; †† *p* < 0.01 or ††† *p* < 0.001 vs. HG + vehicle. The above results are from five independent experiments. (**D**): Representative Western blots and quantitative results of the relative protein levels of p-PDH and PDH in WRL68 cells subjected to HG with the Mu50 Δ*CcpA* or Mu50 strain for 4 h. *** *p* < 0.001 vs. control + vehicle; ††† *p* < 0.001 vs. HG + vehicle. The above results are from five independent experiments. (**E**): Quantitative results of the relative mRNA levels of *PDK1*, *PDK2*, *PDK4*, and *PDHA1* in WRL68 cells subjected to HG with the XN108 Δ*CcpA* or XN108 strain for 4 h. †† *p* < 0.01 vs. HG + vehicle. The above results are from five independent experiments. (**F**): Quantitative results of the relative mRNA levels of *PDK1*, *PDK2*, *PDK4*, and *PDHA1* in WRL68 cells subjected to HG with the Mu50 Δ*CcpA* or Mu50 strain for 4 h. * *p* < 0.05 vs. control + vehicle; † *p* < 0.05 vs. HG + vehicle. The above results are from five independent experiments.

To further clarify the abnormal metabolic phenotype of type 1 diabetic mice caused by *CcpA*-knockout *S. aureus* inhibiting PDH activity by activating the PDH pathway, we used two *S. aureus* strains with different genetic backgrounds for *CcpA* knockout. Moreover, WRL68 hepatocytes induced by high glucose were used to further investigate the molecular mechanism. According to the Western blotting results obtained, although the XN108 Δ*CcpA* strain increased the expression of PDK4 in WRL68 hepatocytes in both normal and high-glucose environments (*p* < 0.01), there was no significant effect observed on either PDK1 or PDK2 (Figure 5A). Nonetheless, the expression of p-PDH (downstream of PDK4) increased notably, particularly in the cells treated with high glucose (*p* < 0.01; Figure 5B).

In addition, compared with the XN108 Δ*CcpA* strain, the Mu50 Δ*CcpA* strain affected not only the expression of PDK4 but also its isozyme PDK2; however, no significant effect on PDK1 was observed. Furthermore, the Mu50 Δ*CcpA* strain may have induced an upregulation effect on the expression of PDK2, PDK4, and p-PDH (all, *p* < 0.001; Figure 5D) in both normal and high-glucose environments.

The RT-PCR results also showed that the XN108 Δ*CcpA* strain may increase the mRNA level of PDK4, but there was no significant effect observed on PDK1, PDK2, or PDHA1 (Figure 5E). Moreover, the Mu50 Δ*CcpA* strain may have increased the expression of PDK2 and PDK4, but no significant effects on that of PDK1 and PDHA1 were observed (Figure 5F).

**Figure 6:**
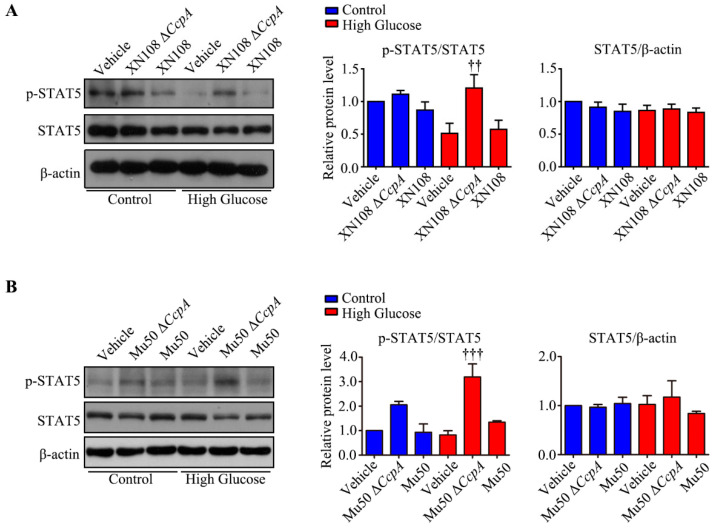
Upstream regulation mechanism of PDK4 under infection with *S. aureus* with different genetic backgrounds in WRL68 hepatocytes induced by high glucose. (**A**): Representative Western blots and quantitative results of the relative protein levels of p-STAT5 and STAT5 in WRL68 cells subjected to HG with the XN108 Δ*CcpA* or XN108 strain for 4 h. †† *p* < 0.01 vs. HG + vehicle. The above results are from five independent experiments. (**B**): Representative Western blots and quantitative results of the relative protein levels of p-STAT5 and STAT5 in WRL68 cells subjected to HG with the Mu50 Δ*CcpA* or Mu50 strain for 4 h. ††† *p* < 0.001 vs. HG + vehicle. The above results are from five independent experiments.

**Figure 7:**
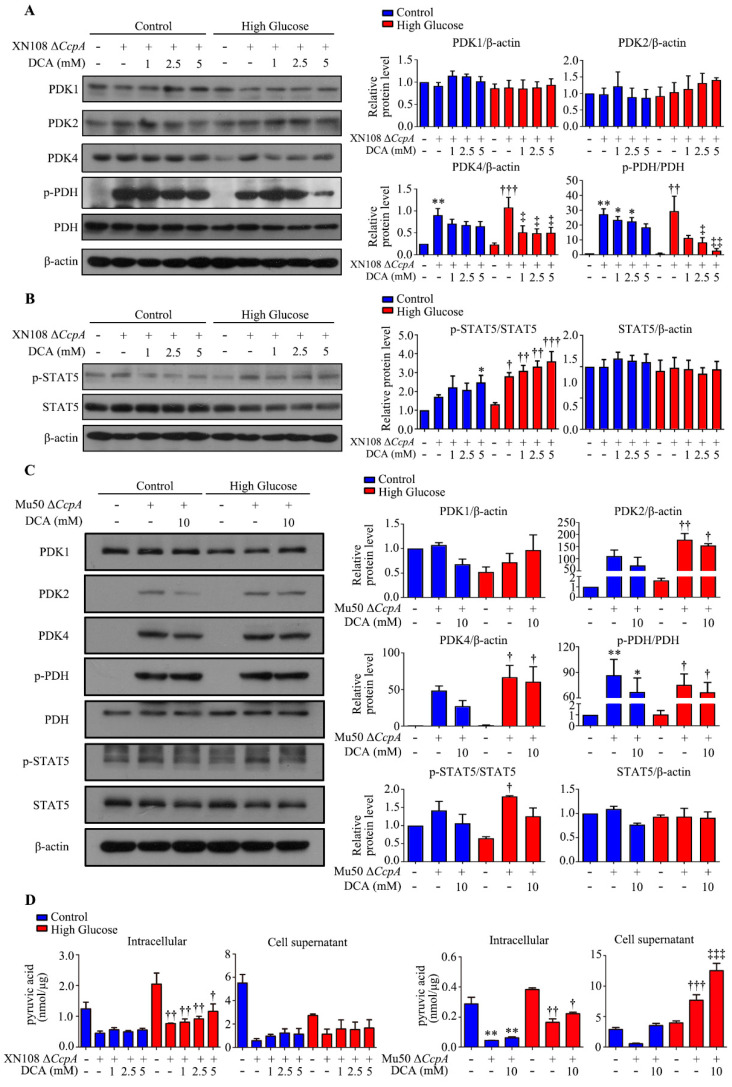
The PDK4 inhibitor sodium dichloroacetate (DCA) restrained the effects of infection with different Δ*CcpA* strains on high-glucose-treated WRL68 hepatocytes. (**A**): Representative Western blots and quantitative results of the relative protein levels of PDK1, PDK2, PDK4, p-PDH, and PDH in WRL68 cells subjected to HG with or without the XN108 Δ*CcpA* strain (4 h) and DCA (12 h before subjecting to HG). * *p* < 0.05 or ** *p* < 0.01 vs. control; †† *p* < 0.01 or ††† *p* < 0.001 vs. HG; ‡ *p* < 0.05 or ‡‡ *p* < 0.01 vs. HG + XN108 Δ*CcpA*. The above results are from five independent experiments. (**B**): Representative Western blots and quantitative results of the relative protein levels of p-STAT5 and STAT5 in WRL68 cells subjected to HG with or without the XN108 Δ*CcpA* strain (4 h) and DCA (12 h before subjecting to HG). * *p* < 0.05 vs. control; † *p* < 0.05, †† *p* < 0.01 or ††† *p* < 0.001 vs. HG. The above results are from five independent experiments. (**C**): Representative Western blots and quantitative results of the relative protein levels of PDK1, PDK2, PDK4, p-PDH, PDH, p-STAT5, and STAT5 in WRL68 cells subjected to HG with or without the Mu50 Δ*CcpA* strain (4 h) and DCA (12 h before subjecting to HG). * *p* < 0.05 or ** *p* < 0.01 vs. control; † *p* < 0.05 or †† *p* < 0.01 vs. HG. The above results are from five independent experiments. (**D**): Quantitative results of the pyruvic acid in the cell supernatant and intracellular fluid of WRL68 cells subjected to HG with or without the XN108 Δ*CcpA* strain (4 h) or Mu50 Δ*CcpA* strain (4 h) and DCA (12 h before subjecting to HG). ** *p* < 0.01 vs. control; † *p* < 0.05 vs. HG + vehicle; †† *p* < 0.01 vs. HG + vehicle; ††† *p* < 0.001 vs. HG + vehicle; ‡‡‡ *p* < 0.001 vs. HG + Mu50 Δ*CcpA*. The above results are from five independent experiments.

The above results reveal that *S. aureus* with *CcpA* knockout may increase the expression of the PDK4 gene and protein in hepatocytes. We used Western blotting to detect the upstream p-STAT5 and identified that the two *S. aureus* strains with *CcpA* knockout with different genetic backgrounds were able to increase p-STAT5 (*p* < 0.01; Figure 6A,B). These results may confirm that *S. aureus* with *CcpA* knockout affects the expression of PDK4 by activating p-STAT5. We then used the PDK4 inhibitor sodium dichloroacetate (DCA) to pretreat the cells with Δ*CcpA S. aureus* for 12 h before infection. The Western blotting results showed that DCA inhibited the increase in Δ*CcpA S. aureus* infection-induced PDK4 expression (Figure 7A–C). Meanwhile, we detected the concentration of pyruvate in the cells and the cell culture medium catalyzed by PDH. The results showed that the concentrations of pyruvate inside and outside the cells decreased significantly after adding the XN108 Δ*CcpA* strain in both the normal and high-glucose environments (Figure 7D). After applying DCA in the high-glucose environment, the concentration of pyruvate in the cells increased, although the difference was not significant (Figure 7D). In addition, DCA showed no significant effect on the concentration of pyruvate in the cell culture medium (Figure 7D). The pyruvate concentration of the Mu50 Δ*CcpA* strain was consistent with that of the XN108 Δ*CcpA* strain in the normal-glucose environment; however, the difference was that the former increased the pyruvate concentration in the cell culture medium in the high-glucose environment (*p* < 0.001). When combined with DCA, the pyruvate concentration in the cell culture medium was lower than that in HG + Mu50 Δ*CcpA* (*p* < 0.001; Figure 7D).

### 3.6. The Secretion of Mu50 ΔCcpA Strains Showed Stimulative Effects on WRL68 Hepatocytes Induced by High Glucose

**Figure 8:**
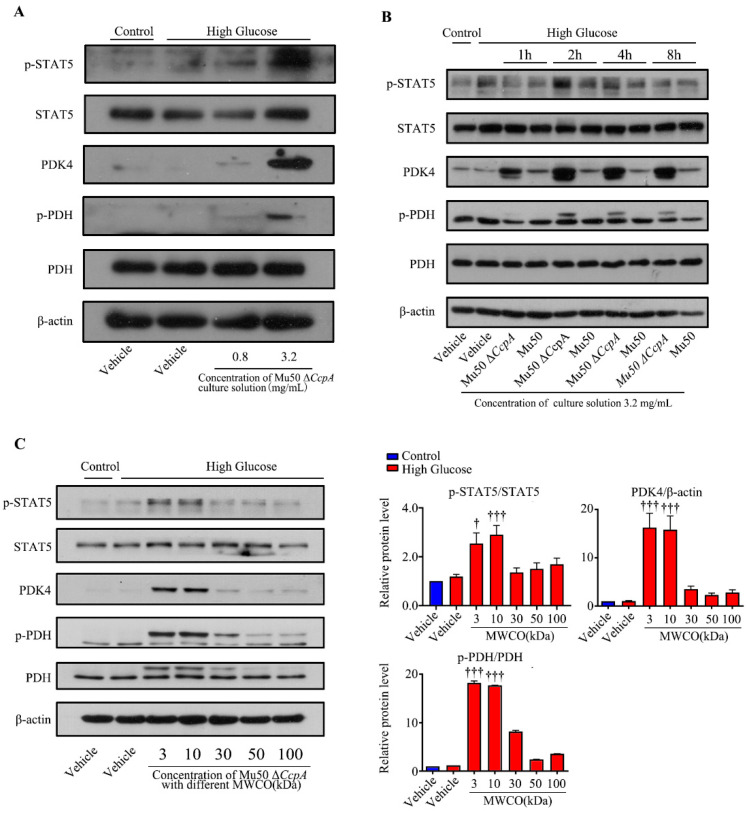
Effects of secretion of the Mu50 Δ*CcpA* strain on WRL68 hepatocytes induced by high glucose. (**A**): Representative Western blots of the relative protein levels of p-STAT5, STAT5, PDK4, p-PDH, and PDH in WRL68 cells subjected to HG with or without concentration (0.8 or 3.2 mg/mL) of the culture solution of the Mu50 Δ*CcpA* strain. (**B**): Representative Western blots of the relative protein levels of p-STAT5, STAT5, PDK4, p-PDH, and PDH in WRL68 cells subjected to HG with concentration (3.2 mg/mL) of culture solution of the Mu50 Δ*CcpA* or Mu50 strain. (**C**): Representative Western blots and quantitative results of the relative protein levels of p-STAT5, STAT5, PDK4, p-PDH, and PDH in WRL68 cells subjected to HG with a concentration of the Mu50 Δ*CcpA* or Mu50 strain with different molecular weight cutoffs. † *p* < 0.05 vs. HG + vehicle; ††† *p* < 0.001 vs. HG + vehicle. The above results are from three independent experiments.

To further understand the reason behind the activation of p-STAT5, we isolated the bacteria of the Mu50 Δ*CcpA* strain and the bacterial culture medium, concentrated the bacterial culture medium, and used the concentrated bacterial culture medium to stimulate WRL68 hepatocytes induced by high glucose. After determining the Mu50 Δ*CcpA* strain concentration of the concentrated medium, we treated the cells with final Mu50 Δ*CcpA* strain concentrations of 0.8 mg/mL and 3.2 mg/mL. The results showed that the p-STAT5/PDK4 pathway was effectively activated under the stimulation conditions with the Mu50 Δ*CcpA* strain at 3.2 mg/mL (Figure 8A). Then, the concentrated medium of the Mu50 wild strain and *CcpA*-knockout strain was used for treatments at 1 h, 2 h, 4 h, and 8 h. The results showed that the concentrated medium of the Mu50 Δ*CcpA* strain (3.2 mg/mL) at 2 h activated the p-STAT5/PDK4 pathway (Figure 8B). Finally, the culture medium of the Mu50 Δ*CcpA* strain was concentrated by centrifuge filtration with different interception sizes (3, 10, 30, 50, and 100 kDa). The filtrates of 3 kDa and 10 kDa were found able to increase the levels of p-STAT5 (*p* < 0.001), PDK4 (*p* < 0.001), and p-PDH (*p* < 0.001) effectively. The results suggest that the Mu50 Δ*CcpA* strain activates the p-STAT5/PDK4 pathway by releasing secretory proteins with a mass in the range of 3–30 kDa (Figure 8C).

## 4. Discussion

After the intravenous injection of *CcpA*-knockout *S. aureus* in type 1 diabetic mice, the random blood glucose level was found to decrease significantly. The blood lipids and serum free fatty acids also increased significantly. Meanwhile, we also found a decrease in the random blood glucose level in type 2 diabetic mice infected with *CcpA*-knockout *S. aureus*. However, the metabolic phenotype changes in type 1 diabetic mice were more obvious, so we focused on type 1 diabetes in this study. The specific mechanisms by which CcpA-knockout *S. aureus* affects type 2 diabetic mice will also be explored in future studies. The results in type 1 diabetic mice suggest that the CcpA protein in the *S. aureus*-infected mice profoundly affected the glucose and lipid metabolism of the host in a high-glucose environment. Furthermore, type 1 diabetic mice and WRL68 human hepatocytes subjected to high-glucose treatments were used as research models to verify that CcpA-knockout *S. aureus* infection activated the signal transducer and activator of the transcription 5 (STAT5)/PDK4 pathway of the liver by secreting proteins, resulting in abnormal metabolic phenotypes of diabetes.

CcpA is a very important global regulatory protein and typically exists in Gram-positive bacteria, such as *S. aureus*, *Streptococcus* spp., and *Bacillus subtilis*. Previous studies have shown that during the exponential growth period of *S. aureus*, more than 150 genes are regulated by CcpA [28]. It is also known that CcpA not only regulates the expression of metabolic genes in *S. aureus* but also controls staphylotoxin secretion, biofilm formation, and antibiotic resistance [29,30]. Considering the important role of CcpA in the virulence of *S. aureus*, it could be a feasible antimicrobial target. The virulence of *S. aureus* may be reduced via chemical inhibition in the interaction of *CcpA* with the Cre DNA region [31]. In the present study, type 1 diabetic mice were treated with *CcpA*-knockout *S. aureus* strains with different genetic backgrounds. After two weeks, evident damage and edema of the hepatocytes in the diabetic mice infected with the wild-type strain XN108 was observed, along with extensive inflammatory cell infiltration in diabetic mice infected with XN108 Δ*CcpA*. The immunohistochemistry results also showed that the expression of inflammatory factors, such as TNF-α and IL-6, decreased significantly in the livers of diabetic mice infected with XN108 Δ*CcpA*, suggesting that the infectivity and pathogenicity of the *CcpA*-knockout *S. aureus* strain XN108 decreased significantly. These results are in accord with previous reports [15]. Furthermore, we reported for the first time that compared with mice infected with wild-type strains, the type 1 diabetic mice infected with XN108 Δ*CcpA* for 2 weeks showed significantly decreased random blood glucose and increased triglyceride and free fatty acid levels, and they also showed obvious metabolic phenotypic changes.

In the present study, RNA was extracted from the liver tissues of each group to investigate the molecular mechanisms behind metabolic phenotypic changes induced by the *S. aureus* Δ*CcpA* strain. The RNA-seq technique was used to analyze the differential expression of genes. It was found that, compared with the diabetic group, the diabetic + Δ*CcpA* group had 1182 differentially expressed genes in the liver, of which 1076 genes were upregulated and 86 genes were downregulated. Our findings suggest that PDK family genes are key target genes for metabolic phenotypic changes. Upon verification in the animal model, we found that infection with the XN108 Δ*CcpA* strain increased the transcription and translation of PDK4 in the liver of type 1 diabetic mice, increased p-PDH expression, and significantly increased the pyruvate level in the tissues.

PDC is a key regulatory enzyme in the second stage of aerobic glucose oxidation and is also known as the hub of aerobic sugar oxidation [16]. It catalyzes the irreversible oxidation of pyruvate to acetyl CoA. PDH is the core structure of PDC. The activity of PDC is regulated by PDK and PDP [17,32]. In the oxidation process, PDH regulates the decarboxylation of pyruvate to hydroxyethyl-TPP. PDK4 phosphorylates and inactivates PDH [24]. Thus, PDK4 inhibits the oxidative decarboxylation of pyruvate in the mitochondria [33]. Consequently, glucose oxidation is inhibited, which leads to glucose utilization disorder. It was interesting to observe that *CcpA*-knockout *S. aureus* infection upregulated PDK4 transcription and translation in the livers of diabetic mice. As a result, PDH phosphorylation was increased, and this led to PDC inactivation. The inactivation of PDC affects the aerobic oxidation of glucose. The reaction process of the oxidative decarboxylation of pyruvate into acetyl CoA is thus limited and results in the accumulation of pyruvate produced by glycolysis, which is consistent with our experimental results.

Furthermore, we studied the upstream mechanism of PDK4 activation induced by XN108 Δ*CcpA* in the livers of diabetic mice. The expression of PDK4 is mainly regulated by the PGC-1α, FOXO1, and JAK2/STAT5 pathways [1,25,34]. Our results showed that the expression of p-STAT5 was significantly upregulated due to XN108 Δ*CcpA* in the livers of diabetic mice. Moreover, the trend of the changes was consistent with that of PDK4, suggesting that XN108 Δ*CcpA* upregulated the expression of PDK4 by activating the STAT5 pathway.

We also used two *S. aureus* strains from different sources and different genetic backgrounds (XN108 and Mu50) to infect the human liver cell line WRL68 induced by high glucose and high glucose combined with the PDK4 inhibitor DCA. This helped further clarify the effect and molecular mechanism of Δ*CcpA S. aureus* on the metabolic phenotype of diabetes and excluded the influence of the genetic background. The results showed that *S. aureus* Δ*CcpA* activated the p-STAT5/PDK4 pathway and caused an increase in PDH phosphorylation and the inactivation of PDH. Conversely, DCA significantly inhibited PDK4, and the phosphorylation of PDH also decreased. The results indicated that p-PDH was increased by PDK4. The significant change in triglycerides caused by infection with *CcpA*-knockout *S. aureus* may be attributed to the accumulation of pyruvate, which promotes the citric acid–pyruvate cycle. Pyruvate can be directly used to synthesize several amino acids. It can also be metabolized into lactic acid or ethyl acetate or converted into acetyl CoA. Acetyl CoA can be metabolized into acetate and introduced into the tricarboxylic acid cycle. It can also be used for the synthesis of fatty acids. Thus, the triglyceride content is increased [35]. However, the results of the cell experiments showed that the Δ*CcpA* strain reduced the concentration of intracellular pyruvate. Moreover, the concentration of pyruvate in the livers of the diabetic + XN108 Δ*CcpA* group was higher than that in the livers of the diabetic + vehicle group. The results of these two parts of the experiment were not consistent. The differences could be attributed to the different strains used in the experiment. The mechanism needs to be further explored for a better understanding.

Taken together, the findings of the cell experiments and the animal experiments may verify that *CcpA*-knockout *S. aureus* infection activates the p-STAT5/PDK4 pathway in the liver. However, how this strain activates p-STAT5 remains unknown. Subsequently, we isolated the cells and culture medium of Mu50 Δ*CcpA*. The bacterial culture medium collected was concentrated and utilized to stimulate WRL cells induced by high glucose. We found that the concentrated culture also activated the STAT5/PDK4 pathway in a concentration- and time-dependent manner. Then, the culture medium was concentrated using a centrifuge filtration device with different interception sizes before stimulating the cells. It was found that secretory proteins with masses 3 kDa and 10 kDa effectively increased the expression of PDK4 in hepatocytes under high-glucose conditions. Therefore, we speculated that Mu50 Δ*CcpA* may activate the STAT5/PDK4 pathway by releasing secretory proteins with a mass of around 30 kDa. Kim et al. reported that growth hormone significantly induced PDK4 expression in the liver through the JAK2/STAT5 signaling pathway. It was reported that growth hormone-mediated PDK4 expression may induce PDC phosphorylation and inhibit pyruvate utilization in the livers of wild-type mice [27]. When glucose levels are low, growth hormone may act as a reverse regulatory hormone of insulin and stimulate adipose tissue to release free fatty acids into the blood and activate the liver JAK2/STAT5 signaling pathway to promote the utilization of free fatty acids in the liver and the surrounding tissues [36]. In this study, we found that the increase in triglyceride and free fatty acid levels in the serum of XN108 Δ*CcpA*-infected diabetic mice was similar to that influenced by growth hormone. We thus speculate that the XN108 Δ*CcpA* strain may activate the STAT5/PDK4 pathway by producing growth hormone-like proteins. The specific types of secreted proteins need to be further identified using protein spectrum analysis and metabolomics. The experimental results may provide new insights for understanding the molecular mechanism underlying glucose and lipid metabolism disorders in patients with diabetes complicated with *S. aureus* infection. The findings of the present study provide vital information for the development of new antimicrobial agents targeting *CcpA* and its use in patients with diabetes.

## Figures and Tables

**Table 1:** Strains and plasmids used in this study.

Strain/Plasmid	Description	Reference
Strains		
*Escherichia coli*		
DH5α	Laboratory strain	Tiangen
*Staphylococcus aureus*		
RN4220	Restriction-deficient cloning host	[1]
Mu50	Vancomycin-intermediate *S. aureus* strain (VISA)	[1]
XN108	A clinical VISA strain	[1]
XN108 Δ*CcpA*	Markerless deletion of *CcpA*	This study
Mu50 Δ*CcpA*	Markerless deletion of *CcpA*	This study
Plasmids		
pBT2	*S. aureus*–*E. coli* shuttle vector, temperature-sensitive, Amp^R^ in *E. coli* and Cm^R^ in *S. aureus*	[1]
pBT2 Δ*CcpA*	pBT2 derivative, for *CcpA* deletion in XN108 and Mu50	This study

## Data Availability

The data that support the findings of this study are available from the corresponding author upon reasonable request.
